# Suppression of Postprandial Blood Glucose Fluctuations by a Low-Carbohydrate, High-Protein, and High-Omega-3 Diet via Inhibition of Gluconeogenesis

**DOI:** 10.3390/ijms19071823

**Published:** 2018-06-21

**Authors:** Bin Wang, Christopher Smyl, Chih-Yu Chen, Xiang-Yong Li, Wei Huang, Hong-Man Zhang, Victor J. Pai, Jing X. Kang

**Affiliations:** 1 Laboratory for Lipid Medicine and Technology (LLMT), Massachusetts General Hospital and Harvard Medical School, Boston, MA 02129, USA; yy_bwang@hotmail.com (B.W.); christopher.smyl@charite.de (C.S.); cchen45@mgh.harvard.edu (C.-Y.C.); xyli75@126.com (X.-Y.L.); whuang_tmmu@hotmail.com (W.H.); hmzhang9607@sina.com (H.-M.Z.); victor.pai@phd.einstein.yu.edu (V.J.P.); 2 Research Center for Nutrition and Food Safety, Institute of Military Preventive Medicine, Third Military Medical University, Chongqing 400038, China; 3 Biomedical Analysis Center, Third Military Medical University, Chongqing 400038, China

**Keywords:** low-carbohydrate high-protein diet, omega-3 fatty acid, hyperglycemia, gluconeogenesis, glycemic control

## Abstract

Hyperglycemia significantly contributes to the development and progression of metabolic diseases. Managing postprandial blood glucose fluctuations is of particular importance for patients with hyperglycemia, but safe and effective means of reducing blood glucose levels are still lacking. Five diets with varying macronutrient ratios and omega-3 fatty acid amounts were tested for their blood glucose-lowering effects in male C57BL/6J mice. The diets with potent blood glucose-lowering effects were further investigated for their underlying mechanisms and their beneficial effects on hyperglycemia models. Mice given the low-carbohydrate, high-protein, and high-omega-3 (LCHP+3) diet exhibited a rapid reduction of the blood glucose levels that remained consistently low, regardless of feeding. These effects were associated with reduced amino acid gluconeogenesis, due to the inhibition of hepatic alanine transaminase (ALT). Furthermore, the LCHP+3 intervention was effective in reducing the blood glucose levels in several disease conditions, including type 1 diabetes mellitus, hormone-induced hyperglycemia, and diet-induced metabolic syndrome. Our findings identify the LCHP+3 diet as a potent blood glucose-lowering diet that suppresses postprandial blood glucose fluctuations through the inhibition of gluconeogenesis and may have great clinical utility for the management of metabolic diseases with hyperglycemia.

## 1. Introduction

Hyperglycemia, a condition in which blood glucose levels are abnormally high, is a central factor in the development of type 2 diabetes mellitus (T2DM) and increases the risk of cardiovascular disease, cancer, and all-cause mortality [1,2,3,4]. Significant postprandial spikes in blood glucose levels generate excess free radicals, leading to inflammation and metabolic dysfunction [5]. Compared with the normal fasting glucose, a doubling of blood glucose levels 2 h after a glucose load is associated with a 1.6-fold increase in cardiovascular event risk [1] and a 2-fold increase in all-cause mortality risk [3]. Currently, glucose-lowering therapies such as anti-diabetic drugs reduce the risk of hyperglycemia and improve the outcomes for patients but have some adverse effects [6,7,8,9,10]. Thus, safe and effective strategies to manage postprandial glucose levels are urgent needed for glycemic control.

Dietary nutrients greatly influence blood glucose levels, and dietary modification is a practical approach to manage diseases with hyperglycemia [11]. In particular, the type and quantity of carbohydrate intake significantly affects blood glucose concentration and insulin secretion [12]. Previous studies have shown that a low-carbohydrate, high-protein dietary intervention can reduce fasting plasma glucose and 24 h glucose response in non-diabetic and T2DM patients [13,14]. However, the intervention outcomes and macronutrient composition of the diets varied between the studies, and the underlying mechanisms of the low-carbohydrate, high-protein diet remain to be evaluated [15]. Given the detrimental impact of the postprandial glucose spike, a diet capable of eliminating or significantly reducing postprandial glucose fluctuations can greatly benefit health conditions requiring glycemic control, such as diabetes and cancer.

Omega-3 polyunsaturated fatty acids (omega-3 PUFA) are known to improve glucose homeostasis, lipid metabolism, inflammatory response, and oxidative stress, and are highly beneficial for patients with diabetes and obesity [16,17,18,19]. In this context, it is possible that the combined use of omega-3 with the LCHP diet can lead a better glycemic control. Therefore, this study was intended to evaluate the effects and mechanism of the combination of a low-carbohydrate, high-protein diet supplemented with high levels of omega-3 PUFA on hyperglycemia.

## 2. Results

### 2.1. The LCHP+3 Diet Has a Rapid and Sustained Lowering Effect on Blood Glucose

**Figure 1:**
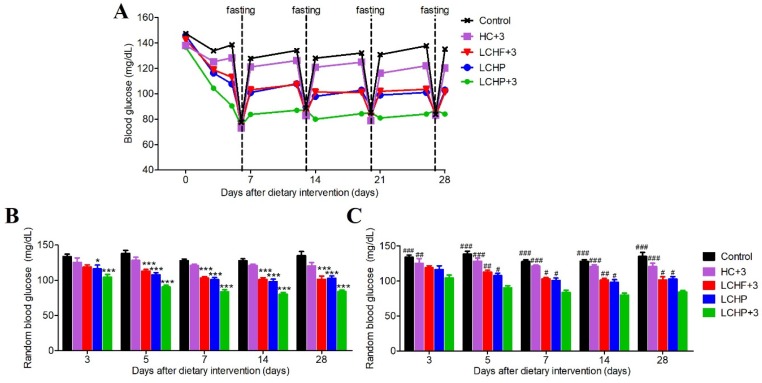
Effects of various experimental diets on blood glucose levels. The five study diets used include a high-carbohydrate/low-omega-3 (Control) diet, a high-carbohydrate/high-omega-3 (HC+3) diet, a low-carbohydrate/high-fat/high-omega-3 (LCHF+3) diet, a low-carbohydrate/high-protein/low-omega-3 (LCHP) diet, and a low-carbohydrate/high-protein/high-omega-3 (LCHP+3) diet (see Tables S1 and S2 for details). (**A**) Random and fasting blood glucose levels at various time points during the feeding duration; (**B**) Representative statistical data for random blood glucose measurements; values are shown as mean ± SEM. Compared with the Control group, * *p* < 0.05, *** *p* < 0.001; (**C**) Representative statistical data for random blood glucose measurements; values are shown as mean ± SEM. Compared with the LCHP+3 group, ^#^ *p* < 0.05, ^##^ *p* < 0.01, ^###^ *p* < 0.001. *n* = 8 per group.

We first set out to compare the effects of five different diets on glucose homeostasis (Control, high-carbohydrate/low-omega-3; HC+3, high-carbohydrate/high-omega-3; LCHF+3, low-carbohydrate/high-fat/high-omega-3; LCHP, low-carbohydrate/high-protein/low-omega-3; LCHP+3, low-carbohydrate/high-protein/high-omega-3; *n* = 8/group). The blood glucose levels were measured at random time points and after overnight fasting (Figure 1A).

Although all three diets low in carbohydrates (LCHF+3, LCHP, LCHP+3) lowered the random blood glucose levels when compared with the Control diet (Figure 1B), the LCHP+3 group exhibited a faster decrease in random blood glucose levels after three days of intervention, and its lowest random blood glucose levels were sustained throughout the experimental period (Figure 1C). Furthermore, unlike the other diet groups, the LCHP+3 group did not exhibit significant postprandial spikes in blood glucose and displayed a stable plateau (around 80–85 mg/dL), regardless of the random or fasting status (Figure 1A). Throughout the study, the body weight and average dietary calories did not differ among the groups (Figure S1, *p* > 0.05). These results indicate that the LCHP+3 diet is able to quickly and effectively lower the blood glucose levels, and that the supplementation of omega-3 to the LCHP diet is instrumental in minimizing postprandial glucose spikes.

### 2.2. The Blood Glucose-Lowering Effect of the LCHP+3 Diet Is Associated with Reduced Amino Acid Gluconeogenesis

**Figure 2:**
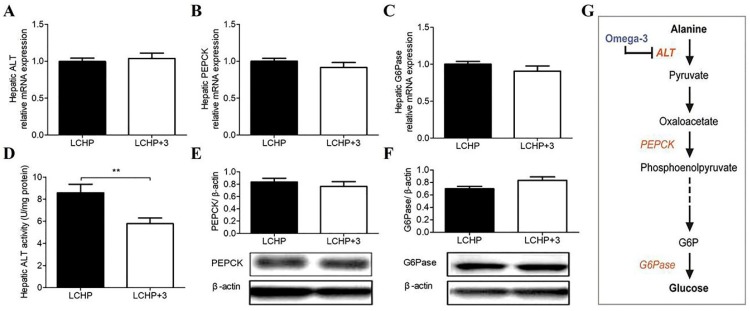
Effects of the LCHP+3 diet on the key enzymes involved in amino acid gluconeogenesis. (**A**) mRNA expression of hepatic ALT; (**B**), mRNA expression of hepatic PEPCK; (**C**) mRNA expression of hepatic G6Pase; (**D**) Hepatic ALT activity; (**E**) Protein expression of hepatic PEPCK; (**F**) Protein expression of hepatic G6Pase; (**G**) Potential target of omega-3 in the amino acid gluconeogenesis pathway. Solid and dashed lines represent one-step and multiple-step reaction, respectively. ALT, alanine aminotransferase; PEPCK, phosphoenolpyruvate carboxy kinase; G6Pase, glucose 6-phosphatase; LCHP, low-carbohydrate/high-protein/low-omega-3; LCHP+3, low-carbohydrate/high-protein/high-omega-3. Values are shown as mean ± SEM; ** *p* < 0.01. *n* = 8 per group.

Next, we chose to compare the effects of the LCHP and LCHP+3 diets to figure out the unique action of omega-3 PUFA in the LCHP background diet and its underlying mechanism (*n* = 8/group). As the conversion of alanine and pyruvate to glucose via the hepatic gluconeogenic pathway is a key mechanism to regulate blood glucose in a low-carbohydrate, high-protein diet, we hypothesized that the LCHP+3 diet was capable of inhibiting hepatic gluconeogenesis from amino acids. The key enzymes in this pathway are: alanine aminotransferase (ALT), the rate-limiting enzyme which catalyzes the conversion of alanine to pyruvate, phosphoenolpyruvate carboxy kinase (PEPCK), which catalyzes the conversion of oxaloacetate to phosphoenolpyruvate, and glucose 6-phosphatase (G6Pase), which catalyzes the ultimate conversion of glucose-6-phosphate to glucose. We first compared the expressions of hepatic *ALT*, *PEPCK*, and *G6Pase* between the two groups, and found no significant differences in the expressions of hepatic *ALT* mRNA and in both mRNA and protein levels of *PEPCK* and *G6Pase* (Figure 2A–C,E,F; *p* > 0.05). However, the hepatic ALT enzymatic activity was significantly lower in the LCHP+3 group than it in the LCHP group (Figure 2D, *p* < 0.01), indicating the potential target of LCHP+3 diet in gluconeogenesis regulation (Figure 2G).

**Figure 3:**
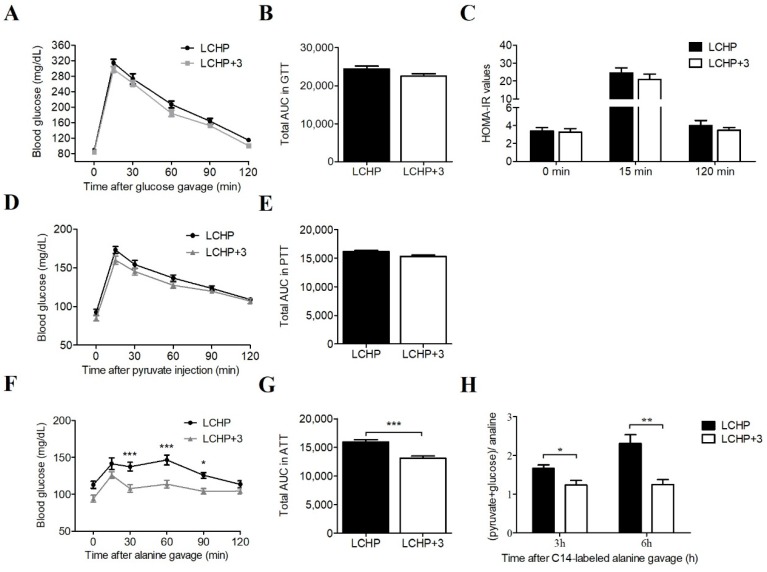
Effects of the LCHP+3 diet on the glycemic control and amino acid gluconeogenesis. (**A**) Blood glucose during GTT, *n* = 12; (**B**) Total AUC in GTT, *n* = 12; (**C**) HOMA-IR values in GTT, *n* = 12; (**D**) Blood glucose during PTT, *n* = 12; (**E**) Total AUC in PTT, *n* = 12; (**F**) Blood glucose during ATT, *n* = 12; (**G**) Total AUC in ATT, *n* = 12; (**H**) ^14^C-radiolabeled alanine conversion, *n* = 6 in each time point. GTT, glucose tolerance test; PTT, pyruvate tolerance test; ATT, alanine tolerance test; AUC, area under the curve; HOMA-IR, homeostasis model assessment of insulin resistance; LCHP, low-carbohydrate/high-protein/low-omega-3; LCHP+3, low-carbohydrate/high-protein/high-omega-3. Values are shown as mean ± SEM; * *p* < 0.05; ** *p* < 0.01; *** *p* < 0.001.

Following this observation, we subsequently conducted a new experiment to explore the regulatory mechanism of the LCHP+3 diet on hepatic gluconeogenesis. Additional twenty-four mice were randomly divided into LCHP and LCHP+3 diet groups (*n* = 12/group). A glucose tolerance test (GTT), the HOMA-IR (homeostasis model assessment of insulin resistance), a pyruvate tolerance test (PTT), and an alanine tolerance test (ATT) were sequentially conducted on the 3rd, 5th, 7th day after dietary intervention, respectively. After 14 days of intervention, we evaluated the effects of the LCHP+3 dietary intervention on protein-derived gluconeogenesis with ^14^C-labeled alanine. Neither the glucose tolerance test (together with HOMA-IR values) nor the pyruvate tolerance test showed significant differences between the two groups, suggesting that the downstream enzymes PEPCK and G6Pase were not involved (Figure 3A–E; *p* > 0.05). Notably, LCHP+3 mice had significantly lower glucose levels than LCHP mice following oral alanine gavage (Figure 3F,G; *p* < 0.001). Furthermore, a ^14^C-radiolabeled alanine challenge experiment indicated that the relative levels of hepatic (pyruvate+glucose)/alanine, a measure of amino acid gluconeogenesis, was significantly lower in the LCHP+3 diet group at both 3 h and 6 h after the ^14^C alanine challenge (Figure 3H; *p* < 0.05 or <0.01; *n* = 6 at each time point). In contrast, the ^14^C radioactivity of alanine in the LCHP+3 group was significantly higher than that in the LCHP group (Figure S2; *p* < 0.05 or <0.01). Collectively, these results suggest that omega-3 PUFA may interact with hepatic ALT to inhibit its activity and reduce amino acid gluconeogenesis.

### 2.3. The LCHP+3 Diet Has Glucose-Lowering Effects in Hyperglycemia Models

**Figure 4:**
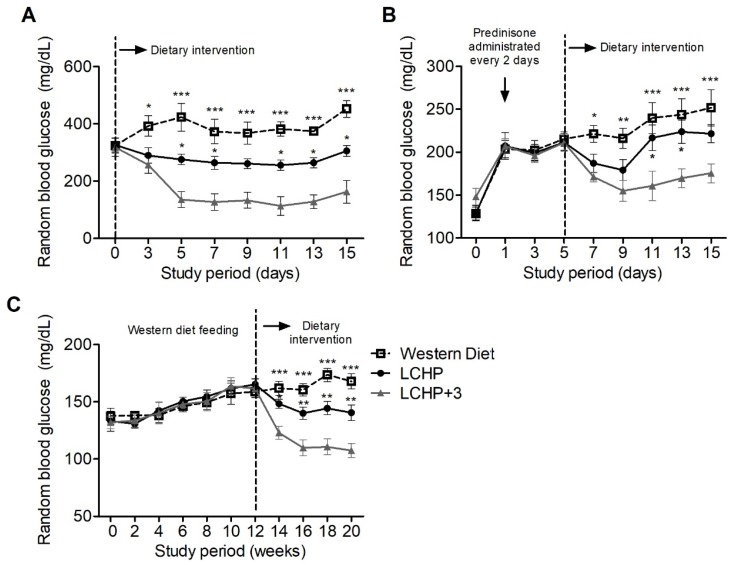
Effects of the LCHP+3 dietary intervention on random blood glucose levels in disease models of hyperglycemia. Three models of hyperglycemia were studied: (**A**) Streptozotocin-induced type 1 diabetes; (**B**) Steroid-induced diabetes-like hyperglycemia; (**C**) Western diet-induced metabolic syndrome. After hyperglycemia induction, the mice were randomly allocated into three groups and fed a control Western diet, the LCHP diet, or the LCHP+3 diet. Values are shown as mean ± SEM. Compared with the LCHP+3 group, * *p* < 0.05, ** *p* < 0.01, *** *p* < 0.001; *n* = 6 per group.

We then explored the potential therapeutic value of the LCHP+3 dietary intervention on various disease conditions with hyperglycemia (*n* = 6/group in each model). Firstly, type 1 diabetes was induced in mice with streptozotocin (STZ), which damages pancreatic β cells and causes insulin deficiency and hyperglycemia [20]. Diabetic mice with random blood glucose around 320 mg/dL were randomly assigned into three groups fed with LCHP, LCHP+3, and Western diets. After three days of intervention, the average random blood glucose of LCHP+3-diet mice was 257 mg/dL (decreased by 19.18%), which was significantly lower than in the Western diet group (Figure 4A, *p* < 0.05). From the fifth day of intervention, the random glucose level in the LCHP+3 group was consistently lower than in the LCHP group (Figure 4A, *p* < 0.05). Throughout the study, no significant difference in average body weight or daily calorie intake was observed among the groups (Figure S3A,B; *p* > 0.05).

Furthermore, steroid-induced diabetes-like hyperglycemia was generated in mice by prednisolone administration, which is known to inhibit the action of insulin and stimulate hepatic gluconeogenesis [21]. We found that 1 h after the first prednisolone administration (day 1), the average blood glucose level was increased by 52.59% when compared with the baseline level at day 0 (Figure 4B; 206 vs. 135 mg/dL). After the third prednisolone administration (day 5), the mice were divided into three groups to further evaluate the effect of the LCHP+3 diet intervention on the steroid-induced hyperglycemia. Our results showed that the hyperglycemia was substantially suppressed in the mice fed the LCHP+3 diet, and the random blood glucose levels were significantly lower than those in the mice fed the Western diet (Figure 4B, *p* < 0.05, <0.01, or <0.001). No statistically significant difference was found between the LCHP-diet and Western diet groups (*p* > 0.05). The average body weight or daily energy intake did not differ among the groups throughout the study (Figure S3C,D; *p* > 0.05). The effectiveness of the LCHP+3 diet in reducing prednisolone-induced hyperglycemia provides further evidence that its effects arise at least in part from the inhibition of gluconeogenesis. 

In addition, we evaluated the effect of the LCHP+3 diet intervention on Western diet-induced chronic hyperglycemia. After the western diet intervention for 12 weeks, the average random blood glucose level was increased by 20.90% (from 134 to 162 mg/dL). In the secondary dietary intervention stage, the mice fed the LCHP+3 diet exhibited marked reductions in the random blood glucose levels, which were significantly lower than in both the LCHP- and Western diet groups (Figure 4C, *p* < 0.05, <0.01 or <0.001). Similar changes were also found in the body weight changes among the groups (Figure S3E). The average daily energy intake did not differ among the groups during the whole study (Figure S3F, *p* > 0.05).

## 3. Discussion

**Figure 5:**
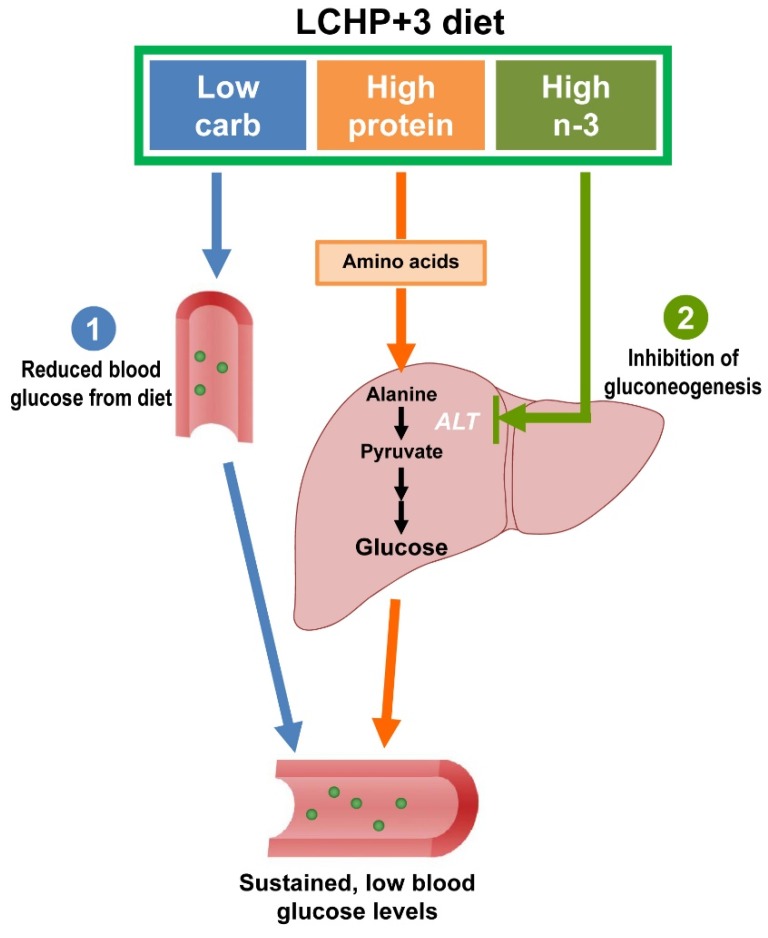
Schema of the potential mechanisms underlying the sustained, blood glucose-lowering effects of the LCHP+3 diet. (1) By reducing exogenous sources of glucose by low-carbohydrate intake; and (2) by inhibiting the endogenous synthesis of glucose through the reduction of protein gluconeogenesis. Blue, orange and green arrows represent the possible role and mechanism of low carbohydrate, high protein and high omega-3 in the glucose-lowering effect of LCHP+3 diet, respectively.

In the present study, we identify the LCHP+3 diet as a potent blood glucose-lowering diet that can minimize the postprandial blood glucose spike and is capable of improving glycemic control in various models of hyperglycemia. The effectiveness of this diet arises from two primary mechanisms: on one hand, the diet reduces exogenous sources of glucose by low-carbohydrate intake, on the other hand, the diet inhibits the endogenous synthesis of glucose by reducing protein gluconeogenesis (Figure 5).

As one of the key enzymes in regulating protein-derived gluconeogenesis [22], ALT is found primarily in the liver, and its serum level is commonly considered a clinical biomarker for liver health. Subsequent studies also demonstrated that high serum ALT is associated with decreased hepatic insulin sensitivity and increased risk of T2DM [23,24]. In addition, it was reported that an omega-3 PUFA supplement could improve serum ALT levels in patients with nonalcoholic fatty liver disease [25]. However, as far as we know, no previous studies have directly evaluated the association between the glucose-lowering effect of omega-3 and hepatic ALT regulation. In this study, our results of the hepatic ALT enzymatic activity, alanine tolerance test, and ^14^C-radiolabeled alanine conversion consistently support the notion that the inhibition of hepatic ALT in the gluconeogenic pathway by omega-3 PUFA is the primary mechanism underlying the sustained, blood glucose-lowering effects of the LCHP+3 diet. In addition, the facts that the effects of the LCHP+3 diet take place quickly within a few days of feeding and hepatic ALT mRNA and protein expression are not altered suggest that omega-3 PUFA may act directly on the hepatic ALT enzyme rather than downregulating its expression. Taken together, these results suggest a potential novel mechanism for the beneficial effects of omega-3 on glycemic control, although it needs to be further elucidated.

Constant hyperglycemia plays a central role in the pathogenesis of diabetes and its associated complications, which are major causes of disability and mortality worldwide [26,27]. Compared with the current glucose-lowering drug therapies with narrow risk–benefit windows, a dietary adjustment for glycemic control shows more promise. Owing to the fact that dietary carbohydrates are the key element in eliciting the postprandial glucose response, there has been growing interest in evaluating the effect of carbohydrate restriction on hyperglycemia management [13,28,29,30]. A recent systematic review based on 26 studies also suggested that a low-carbohydrate diet has beneficial effects on glycemic control and body weight control in T2DM patients [14]. Consistently, our study showed that the random glucose levels in all three low-carbohydrate diet groups (LCHF+3, LCHP, and LCHP+3) were significantly lower than in the Control diet group. However, it should be noted that only the mice fed the LCHP+3 diet had low and stable blood glucose levels in both normal status and hyperglycemia models. More importantly, given that omega-3 PUFA are also known to have beneficial effects on the regulation of hyperlipidemia, oxidative stress, and inflammation in T2DM patients [18,31,32,33], the LCHP+3 diet is an ideal treatment approach for these metabolic disorders and can also comprehensively improve their diverse pathologies and prognosis. 

While it can be expected that a lower carbohydrate intake results in lower blood glucose levels, the other unique effect of the LCHP+3 diet compared to the other low carbohydrate diets was its suppression of blood glucose fluctuations (the glucose levels were maintained stable between 80–85 mg/dL in both random and fasting status). Accumulating data have demonstrated that an acute blood glucose fluctuation can increase oxidative stress and aggravate the inflammatory response, which play important roles in the subsequent vascular damage and pathogenesis of diabetes complications [34,35,36]. Substantial evidence also suggested that daily glucose fluctuations exhibited a more specific triggering effect on oxidative stress than chronic sustained hyperglycemia [37]. Therefore, it has been proposed that minimal blood glucose variability should be the gold standard for evaluating the effectiveness of glycemic control [38]. In this context, the LCHP+3 diet could be a low-cost, accessible, non-drug intervention with quick glucose-lowering and fluctuation-suppressing effects for patients, especially those who need to limit insulin secretion (e.g., diabetes and cancer patients) or those who cannot tolerate other drug treatments. 

Although our study provides substantial evidence of the novel effects of the LCHP+3 diet on glucose homeostasis, some caution should be taken when interpreting and applying this dietary model to clinical situations. Firstly, despite growing epidemiological data support the beneficial effect of the LCHP diet on human health, there is currently no scientific consensus on the standard definition of a low-carbohydrate diet (from ketogenic diet to 45% of total energy) [39]. We designed the macronutrient ratio of the LCHP+3 diet as 15:60:25 (carbohydrate/protein/fat, % of energy), to avoid an extremely low-carbohydrate intake, which may lead to poor compliance. Nevertheless, the optimal dietary carbohydrate amount needs to be further investigated to ensure both effectiveness and compliance. Secondly, despite previous studies have demonstrated that the LCHP diet has potential beneficial effects on the management of obesity, T2DM, and cardiovascular diseases (CVD) [13,35,36,40], the long-term effect and safety of a high-protein diet consumption still needs to be well elucidated. Thirdly, previous epidemiological studies evaluating the effects of the LCHP diet or omega-3 supplementation on chronic diseases have generated inconsistent results, possibly due to the diverse sources, categories, and qualities of nutrients that may generate potential variations. Therefore, more well-designed and long-term interventional studies are warranted to validate the efficacy of the LCHP+3 diet and optimize the intervention strategy.

To our knowledge, this is the first study to demonstrate that the LCHP diet combined with omega-3 PUFA supplementation can effectively lower blood glucose and minimize postprandial blood glucose fluctuations and is also effective in controlling hyperglycemia in several disease conditions. Our results also identify a new mechanism by which omega-3 PUFA may reduce amino acid gluconeogenesis by inhibiting hepatic ALT enzyme activity. These findings provide compelling evidence for the use of the LCHP+3 diet in the acute management of glycemic control, particularly for patients with severe hyperglycemia.

## 4. Materials and Methods

### 4.1. Animals and Diet Design

All animal procedures were approved by the Institutional Animal Care and Use Committee (IACUC) of Massachusetts General Hospital (Protocol No. 2010N000038; latest approval date: 3/11/2016). Eight-week-old male C57BL/6J mice were purchased from Jackson Laboratory (Bar Harbor, ME, USA). All mice were housed in pathogen-free standard cages with access to water and food and underwent a three-day acclimatization period before the dietary intervention. The body weight was measured once a week, and the average food intake was calculated every three days, unless otherwise specified. The mice were sacrificed following an overnight fast. Blood samples were collected and centrifuged, and tissues were harvested for histological examination or frozen for future analysis.

The five study diets consisted of Control diet, HC+3 diet, LCHF+3 diet, LCHP diet, and LCHP+3 diet (Table S1). These diets were chosen in order to compare the glycemic control effects of diets with different macronutrient compositions, with or without omega-3 PUFA. Each diet had a “high” energy contribution percentage (~60%) from one of the three major macronutrients (carbohydrate, protein, or fat), on the basis of diets commonly used for dietary studies. The fatty acid profiles of the diets were analyzed using an Agilent 6890N (Agilent Technologies, Palo Alto, CA, USA) as described previously [41] and are presented in Table S2. In the parallel studies, the D12079B Western diet (Research Diets, New Brunswick, NJ, USA) was used to induce or exacerbate the different hyperglycemia models (Table S3).

### 4.2. Measurement of Blood Glucose and Glucose Homeostasis

Blood glucose was measured using Contour glucose meter and test strips (Bayer, Elkhart, IN, USA) in blood collected from the tail tip. Random blood glucose was measured from 2:00 to 3:00 p.m., and fasting blood glucose was measured at 9:00 a.m., after a 15 h overnight fast. GTT was performed after overnight fasting, measurement of body weight (BW), and administration of 20% d-glucose solution (2 g/kg BW) by oral gavage. The blood glucose levels were measured at 0, 15, 30, 60, 90, and 120 mins after glucose administration. Insulin secretion was analyzed during the GTT from blood collected at 0, 15, and 120 min. The plasma insulin levels were measured by ELISA (Millipore, Billerica, MA, USA). Homeostasis model assessment of insulin resistance (HOMA-IR) levels were determined using the following formula: HOMA-IR = [glucose (mmol/L) × insulin (μ IU/mL)]/22.5, as described in an earlier study [42]. For PTT and ATT, the mice were fasted for 6 h and injected intraperitoneally with pyruvate (Sigma-Aldrich, St. Louis, MO, USA; 2 g/kg BW) or orally gavaged with L-alanine (Sigma-Aldrich; St. Louis, MO, USA; 2 g/kg BW) [43]. The blood glucose levels were measured at 0, 15, 30, 60, 90, and 120 min after pyruvate administration. The glucose response to GTT, PTT, or ATT was calculated as the area under the curve (AUC), using the linear trapezoidal formula.

### 4.3. Real-Time RT-PCR Analysis

Total RNA was isolated from the liver using Trizol (Invitrogen, Carlsbad, CA, USA), and cDNA was prepared with total RNA (2 μg) using the Reverse Transcription System Kit (Promega, Madison, WI, USA). The PCR primers for ALT, PEPCK, and G6Pase were obtained from PrimerBank (Massachusetts General Hospital, Boston, MA, USA). PCR was performed in duplicate using SYBR green PCR Master Mix and the StepOnePlus Real-Time PCR System (Applied Biosystems, Carlsbad, CA, USA).

### 4.4. Immunoblot Analysis

Total protein extracts of liver tissue were homogenized, prepared using RIPA buffer, and measured for protein, using a BCA kit (Pierce, Rockford, IL, USA). Proteins (100 μg) were separated on a 4–12% SDS–PAGE gel and transferred to a PVDF membrane (Invitrogen, Carlsbad, CA, USA). The primary antibodies, including rabbit anti-PEPCK (1:1000), goat anti-G6Pase (1:500), and mouse anti-β-actin (1:2000), were obtained from Santa Cruz Biotechnology (Santa Cruz, CA, USA). The secondary antibodies included horseradish peroxidase-conjugated ECL goat anti-rabbit IgG, rabbit anti-goat IgG, and goat anti-mouse IgG (1:3000; Santa Cruz Biotechnology). Chemiluminescent detection was performed using the ECL method (Santa Cruz Biotechnology). The band intensities were quantified using ImageJ software 6.0 (NIH, Bethesda, MD, USA).

### 4.5. Hepatic ALT Assay and Determination of Gluconeogenesis with [^14^C]-Alanine

The hepatic ALT activity was measured to assess the effects of different dietary compositions on protein-derived gluconeogenesis [22]. Aliquots of liver tissue (50 mg) were homogenized in cold Tris buffer (100 mmol/L, pH 7.8) and centrifuged at 10,000× *g* for 15 min at 4 °C. ALT activity was measured using the Alanine Transaminase Activity Assay Kit (Cayman, Ann Arbor, MI, USA), and the final hepatic ALT activities were corrected by the respective protein concentrations of the liver samples.

Protein-derived gluconeogenesis was determined using ^14^C-labeled alanine. The mice were fed the LCHP or LCHP+3 diets, fasted overnight for 15 h, and the diets were reintroduced (3 g/mouse) for 2 h. The mice were then administered 2 µCi of L-[^14^C(U)] alanine (Perkin Elmer, Norwalk, CT, USA) by oral gavage. The mice were euthanized at 3 or 6 h after gavage, and the liver tissues were excised immediately. The isolation of hepatic alanine, glucose, and pyruvate were performed as previously described with minor modifications [44,45]. In brief, liver tissue (200 mg) was homogenized and deproteinized using 1.2 mL 10% HClO_4_. Two-thirds of the HClO_4_ extracts were reacted with one-tenth of the volume of 2:4-dinitrophenylhydrazine (0.2% in 2 M HCl) for 30 min and then extracted with ethyl acetate, followed by 10% Na_2_CO_3_ extraction. The extracts were chilled in concentrated HCl for acidification and ethyl acetate re-extraction and finally dried over anhydrous Na_2_SO_4_ for labeled pyruvate isolation (all procedures were performed in the dark at 0 °C). The remaining HClO_4_ extracts were neutralized with 600 μL saturated KHCO_3_ and then passed sequentially through Dowex-50 and Dowex-1 ion-exchange columns (Bio-Rad, Hercules, CA, USA). The labeled glucose and alanine were eluted by 5 mL of deionized water and 5 mL of 2N NH_4_OH, respectively. All isolates were blow-dried under nitrogen, re-dissolved in 100 μL deionized water, and measured using the Tri-Carb 2900TR Liquid Scintillation Analyzer (PerkinElmer, Boston, MA, USA).

### 4.6. Streptozotocin-Induced Diabetes Model

Diabetes was induced in mice by STZ and Western diet following the protocol of Bellenger et al. [20] with minor modifications. Briefly, STZ (Cayman Chemical, Ann Arbor, MI, USA) was dissolved in 0.1 M sodium citrate buffer (pH 4.0) and administered via intraperitoneal injection. The mice received 50 mg/kg/day for five consecutive days, and their blood glucose levels were measured every day after the fifth STZ injection. Mice with non-fasting blood glucose levels exceeding 300 mg/dL were considered diabetic and subsequently randomly divided into three groups for dietary intervention (Western diet, LCHP diet, and LCHP+3 diet). After three days of dietary intervention, the blood glucose levels were monitored every other day.

### 4.7. Steroid-Induced Hyperglycemia Model

The mice were fed the Western diet during the modeling stage of hyperglycemia. Every other day throughout the experiment, the mice were orally gavaged with two doses of 55 mg/kg of prednisolone phosphate (Cayman Chemical) at an interval of 20 min. The blood glucose levels were measured 2 h after the second gavage. After the third prednisolone administration, the mice were randomly allocated into three groups and fed the Western diet, LCHP diet, or LCHP+3 diet.

### 4.8. Western Diet-Induced Metabolic Syndrome Model

The mice were fed the Western diet for 12 weeks to induce metabolic syndrome and chronically high levels of blood glucose. After this, the mice were randomly allocated into three groups and fed the Western diet, LCHP diet, or LCHP+3 diet for additional eight weeks. Random blood glucose, body weight, and average food intake in the groups were measured every two weeks.

### 4.9. Statistical Analysis

All data are presented as means plus or minus standard error of the mean (±SEM). GraphPad Prism 6.0 (GraphPad Software Inc., La Jolla, CA, USA) was used for statistical analysis. Differences among the groups were analyzed using one-way analysis of variance (ANOVA) followed by Bonferroni’s post hoc test. Differences between two groups were evaluated using the unpaired Student’s t test. A *p*-value of <0.05 was considered statistically significant.

## 5. Conclusions

In conclusion, our study has demonstrated that the combination of omega-3 fatty acids with a low-carbohydrate, high-protein diet not only lowers the blood glucose levels more effectively than the other diets, but also has the unique effect of minimizing the postprandial blood glucose spike. The novel findings from this study suggest that the glucose-lowering effect of omega-3 fatty acids is background diet-dependent. Furthermore, our study also provides new insights into the role of omega-3 fatty acids in controlling severe hyperglycemia and diabetes complications.

## Supplementary Materials

Supplementary materials can be found at http://www.mdpi.com/1422-0067/19/7/1823/s1.

## Abbreviations

ALT	Alanine aminotransferase
ATT	Alanine tolerance test
G6Pase	Glucose 6-phosphatase
GTT	Glucose tolerance test
HOMA-IR	Homeostasis model assessment of insulin resistance
ITT	Insulin tolerance test
LCHP+3	Low-carbohydrate, high-protein and high-omega-3 diet
MUFA	Monounsaturated fatty acid
PEPECK	Phosphoenolpyruvate carboxy kinase
PTT	Pyruvate tolerance test
PUFA	Polyunsaturated fatty acid
SFA	Saturated fatty acid
STZ	Streptozotocin
