# Supplementary material for: Suppression of Postprandial Blood Glucose Fluctuations by a Low-Carbohydrate, High-Protein, and High-Omega-3 Diet via Inhibition of Gluconeogenesis
Source: Int J Mol Sci. 2018 Jun 21;19(7):1823. doi: 10.3390/ijms19071823 (PMC6073393; doi:10.3390/ijms19071823)
Supplement: Supplementary file 1 [file ijms-19-01823-s001.pdf]

Supplementary Materials

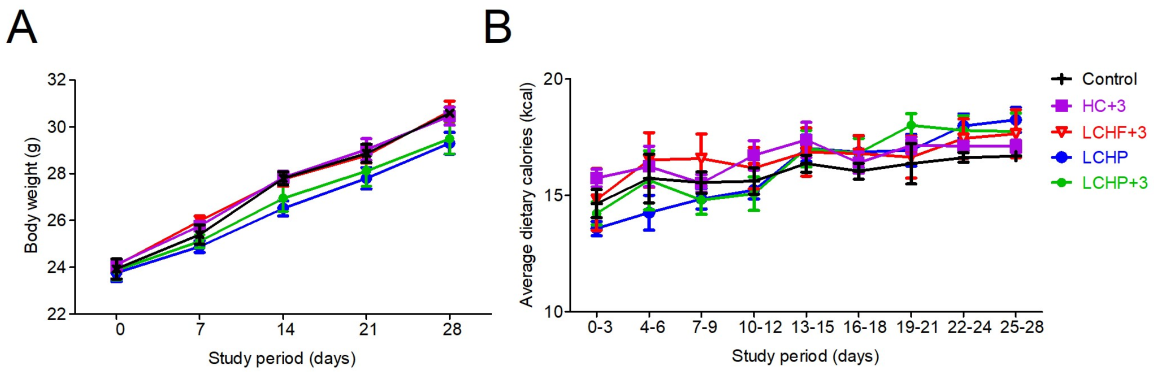

Figure S1. Effects of various diets on body weight and dietary energy intake during the whole study period. (**A**) Body weight; (**B**) Dietary energy intake. The five study diets consisted of a high carbohydrate/low omega-3 (Control) diet, a high carbohydrate/high omega-3 (HC+3) diet, a low carbohydrate/high fat/high omega-3 (LCHF+3) diet, a low carbohydrate/high protein/low omega-3 (LCHP) diet, and a low carbohydrate/high protein/high omega-3 (LCHP+3) diet (see Tables S1 and S2 for details). Values shown as mean ± SEM. $n = 8$ per group.

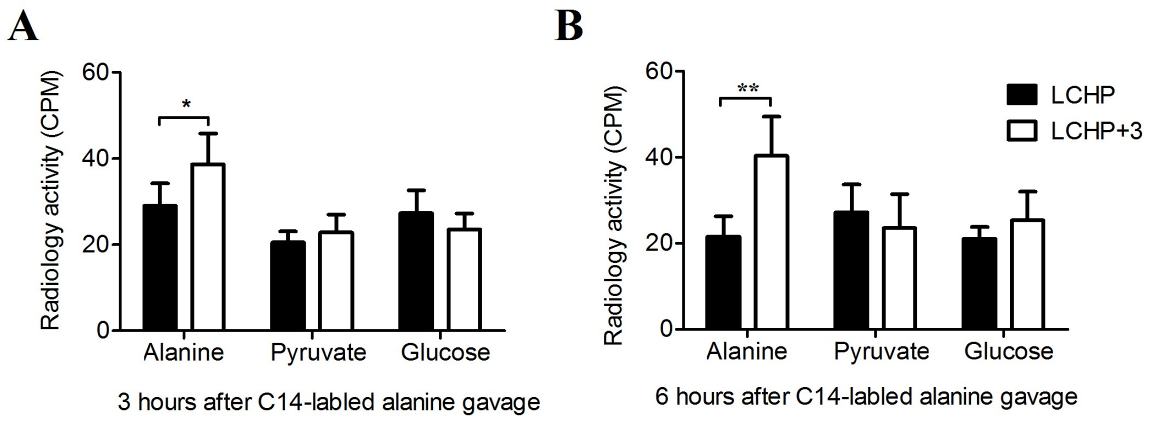

Figure S2. Effects of the LCHP+3 diet on ^{14}C-radiolabeled alanine conversion. (**A**) 3 hours after ^{14}C-alanine gavage; (**B**) 6 hours after ^{14}C-alanine gavage. LCHP, low carbohydrate/high protein/ low omega-3; LCHP+3, low carbohydrate/high protein/high omega-3. Values shown as mean ± SEM; * $p < 0.05$, ** $p < 0.01$; $n = 6$ per group.

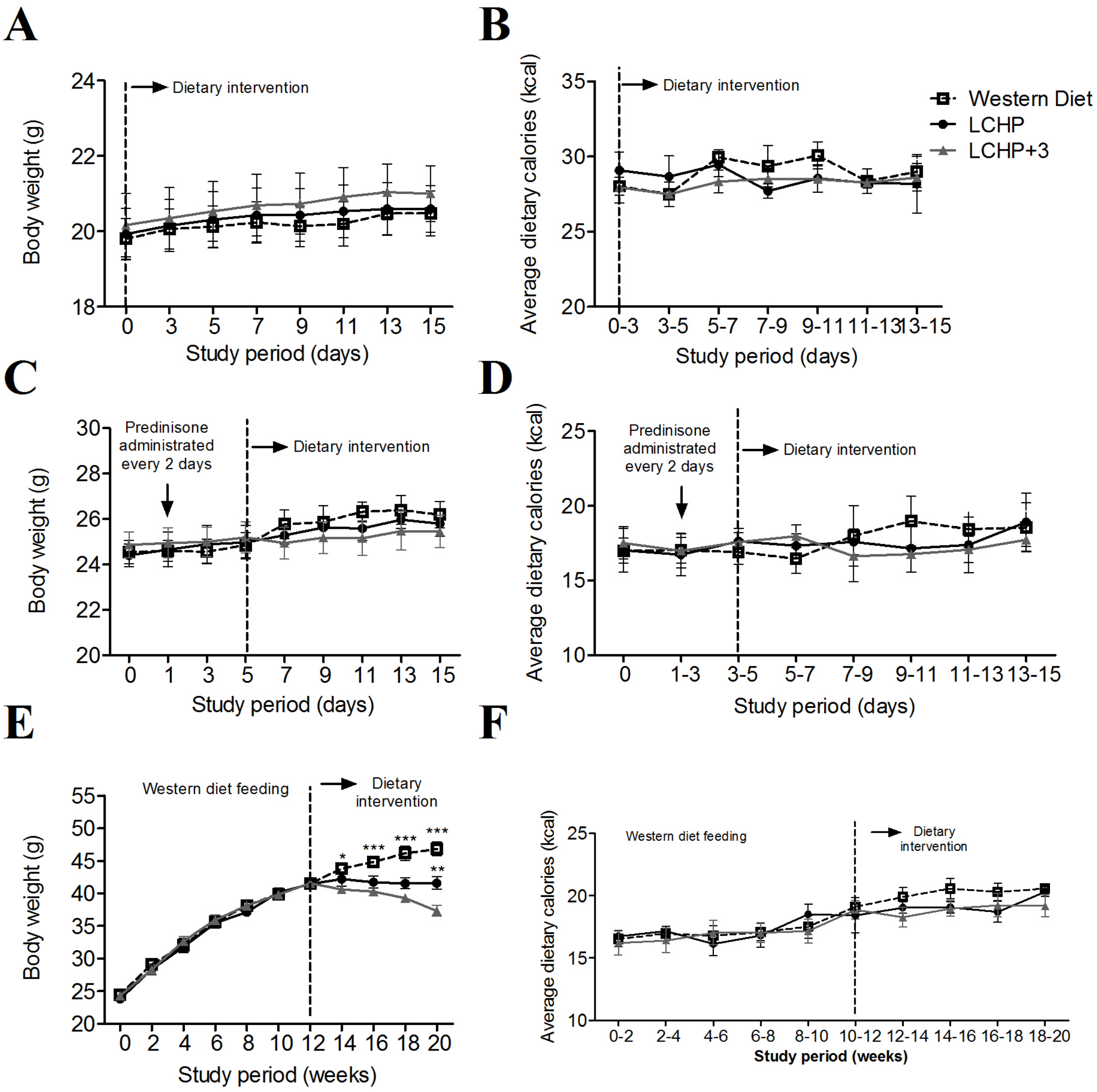

Figure S3. Changes of body weight and energy intake among the groups during the study period of various hyperglycemia models. (**A**,**B**) Streptozotocin-induced type 1 diabetes; (**C**,**D**) Steroid-induced diabetes-like hyperglycemia; (**E**,**F**) Western diet-induced metabolic syndrome. LCHP, low carbohydrate/high protein/low omega-3; LCHP+3, low carbohydrate/high protein/high omega-3. Values shown as mean ± SEM. Compared with the LCHP+3 group, * $p < 0.05$, ** $p < 0.01$, *** $p < 0.001$; $n = 6$ per group.

Table S1. Nutrient composition of the experimental diets (per 100g diet).

	Control	HC+3	LCHF+3	LCHP	LCHP+3
**Carbohydrates (g)**	63	63	20	16	16
Corn starch	15.75	15.75	5	4	4
Sucrose	47.25	47.25	15	12	12
**Protein (g)**	15.3	15.3	34.3	62.3	62.3
Casein	15	15	34	62	62
DL-Methionine	0.3	0.3	0.3	0.3	0.3
**Fat (g)**	12	12	36	12	12
Fish oil	--	4	12	--	4
Corn oil	5	1	3	5	1
Coconut oil	2	2	11	2	2
Flax oil	1	2	6	1	2
Cow butter	4	3	4	4	3
Cellulose	5	5	5	5	5
Mineral Mix S10001	3.5	3.5	3.5	3.5	3.5
Vitamin Mix V10001	1	1	1	1	1
Choline Bitartrate	0.2	0.2	0.2	0.2	0.2
**Total (g)**	100	100	100	100	100
**Energy contribution**					
Carbohydrate (kcal %)	59.8	59.8	14.8	15.2	15.2
Protein (kcal %)	14.5	14.5	25.4	59.2	59.2
Fat (kcal %)	25.6	25.6	59.9	25.6	25.6
kcal/g diet	4.21	4.21	5.41	4.21	4.21

Control, high carbohydrate/low omega-3; HC+3, high carbohydrate/high omega-3; LCHF+3, low carbohydrate/high fat/high omega-3; LCHP, low-carbohydrate/high-protein/low omega-3; LCHP+3, low-carbohydrate/high-protein/high omega-3.

Table S2. Fatty acid profile of the experimental diets (% of total fatty acids).

	Control	HC+3	LCHF+3	LCHP	LCHP+3
**SFA (%)**	43.63	37.84	41.77	43.63	37.84
C8:0	0.62	0.52	0.59	0.62	0.52
C10:0	9.70	9.42	16.10	9.70	9.42
C12:0	8.39	7.50	9.18	8.39	7.50
C16:0	18.71	13.78	10.59	18.71	13.78
C17:0	0.26	0.26	0.16	0.26	0.26
C18:0	5.42	5.79	4.68	5.42	5.79
C20:0	0.17	0.32	0.31	0.17	0.32
**MUFA (%)**	23.13	17.10	13.99	23.13	17.10
C14:1	0.47	0.35	0.16	0.47	0.35
C16:1	0.83	1.18	0.87	0.83	1.18
C17:1	0.13	0.19	0.14	0.13	0.19
C18:1	21.47	14.58	12.06	21.47	14.58
C20:1	0.20	0.79	0.75	0.20	0.79
C22:1	0.03	0.01	0.01	0.03	0.01
**PUFA (%)**	33.24	45.06	44.23	33.24	45.06
**omega-6 PUFA (%)**	27.89	10.88	10.16	27.89	10.88
C18:2	26.58	9.73	9.03	26.58	9.73
C18:3 (omega-6)	1.31	0.33	0.31	1.31	0.33
C20:4	--	0.82	0.82	--	0.82
**omega-3 PUFA (%)**	5.35	34.18	34.07	5.35	34.18
C18:3 (omega-3)	5.35	9.98	9.87	5.35	9.98
C20:5	--	14.08	14.08	--	14.08
C22:5	--	1.66	1.66	--	1.66
C22:6	--	8.46	8.46	--	8.46
**omega-6/omega-3 ratio**	5.21	0.32	0.30	5.21	0.32

Control, high carbohydrate/low omega-3; HC+3, high carbohydrate/high omega-3; LCHF+3, low carbohydrate/high fat/high omega-3; LCHP, low-carbohydrate/high-protein/low omega-3; LCHP+3, low-carbohydrate/high-protein/high omega-3; SFA, saturated fatty acid; MUFA, monounsaturated fatty acid; PUFA, polyunsaturated fatty acid.

Table S3. Macronutrient and omega-6/omega-3 ratios of the diets used in the parallel studies.

	LCHP Diet	LCHP+3 Diet	Western Diet
# Product	Self-made	Self-made	ResearchDiets (D12079B)
Energy contribution			
Carbohydrate (% kcal)	25.6	25.6	43.0
Protein (% kcal)	59.2	59.2	17.0
FAT (% kcal)	15.2	15.2	41.0
kcal/g	4.21	4.21	4.69
omega-6/omega-3 ratio	5.21	0.32	11.04

LCHP, low-carbohydrate/high-protein/low omega-3; LCHP+3, low-carbohydrate/high-protein/high omega-3.
